# EP_2_ receptor antagonism reduces peripheral and central hyperalgesia in a preclinical mouse model of endometriosis

**DOI:** 10.1038/srep44169

**Published:** 2017-03-10

**Authors:** Erin Greaves, Andrew W. Horne, Helen Jerina, Marta Mikolajczak, Lisa Hilferty, Rory Mitchell, Sue M. Fleetwood-Walker, Philippa T. K. Saunders

**Affiliations:** 1 MRC Centre for Reproductive Health, The University of Edinburgh, Queen’s Medical Research Institute, 47 Little France Crescent, Edinburgh EH16 UK; 2 Centre for Integrative Physiology, The University of Edinburgh, Hugh Robson Building, 15 George Square, Edinburgh EH8 UK; 3 MRC Centre for Inflammation Research, The University of Edinburgh, Queen’s Medical Research Institute, 47 Little France Crescent, Edinburgh EH16 UK.

## Abstract

Endometriosis is an incurable gynecological disorder characterized by debilitating pain and the establishment of innervated endometriosis lesions outside the uterus. In a preclinical mouse model of endometriosis we demonstrated overexpression of the PGE_2_-signaling pathway (including COX-2, EP_2_, EP_4_) in endometriosis lesions, dorsal root ganglia (DRG), spinal cord, thalamus and forebrain. TRPV1, a PGE_2_-regulated channel in nociceptive neurons was also increased in the DRG. These findings support the concept that an amplification process occurs along the pain neuroaxis in endometriosis. We then tested TRPV1, EP_2_, and EP_4_ receptor antagonists: The EP_2_ antagonist was the most efficient analgesic, reducing primary hyperalgesia by 80% and secondary hyperalgesia by 40%. In this study we demonstrate reversible peripheral and central hyperalgesia in mice with induced endometriosis.

Endometriosis is a chronic gynecological disorder affecting 176 million women worldwide^1^ associated with chronic pain and infertility. Current therapies include invasive surgery, drugs that suppress endogenous hormones^2^ and non-steroidal anti-inflammatory drugs (NSAIDs) all of which have unwanted side effects. Better treatments for endometriosis-associated pain are needed but their development has been hampered by the lack of a robust preclinical model that fully reproduces the altered pain perception experienced by women with endometriosis.

Endometriosis is caused by the presence of endometrial-like tissue (endometriosis lesions) outside of the uterine cavity^3^. An association between small nerve fiber infiltration of endometriosis lesions and increased central and peripheral pain has been described (reviewed in ref. 4). Alterations in pain perception in women are thought to involve release of inflammatory mediators and neuropeptides by efferent peripheral nerve endings^5^, an increase in the sensitivity of nociceptive neurons^6^, and peripheral hyperalgesia. Continuous input from peripheral afferents can also trigger spinal hyper-excitability (central sensitization)^7^ resulting in increased pain perception, secondary hyperalgesia and allodynia^8^. Prostaglandin E_2_ (PGE_2_) is a well-established mediator of inflammation and nociception in inflammatory^9^,^10^ and neuropathic pain conditions^11^ and synthesis of prostaglandins within endometriosis lesions has previously been reported^12^,^13^,^14^,^15^. As the association of PGE_2_ signaling and endometriosis is well established we chose to validate this pathway as a target for pain attenuation in our mouse model.

A rat model of endometriosis exhibits vaginal hyperalgesia and increased abdominal muscle activity^16^,^17^,^18^. Recently, a study utilizing the same rat model demonstrated increased hyperalgesia in response to von Frey filaments, although an increase was also observed in the sham group^19^. Another model using autologous transplantation of endometrial tissue onto the gastrocnemius muscle^20^ has proved useful for the identification of pronociceptive molecules that may be relevant in endometriosis^21^. Our murine model of endometriosis uses decidualized endometrial tissue injected (rather than sutured) into the peritoneum of recipient mice^22^. Resultant lesions phenocopy those in women; they are vascularized, innervated, infiltrated by macrophages and also exhibit oestrogen-dependent regulation of vascular-nerve and macrophage-nerve interactions^23^,^24^. The aim of our study was to determine if mice with induced endometriosis exhibit peripheral (abdominal) and central (secondary/referred) hyperalgesia and to test compounds targeting prostaglandin receptors to validate this model as a platform for preclinical testing of compounds to treat pain.

## Results

### Induction of endometriosis lesions resulted in altered pain-associated behaviors

**Figure 1:**
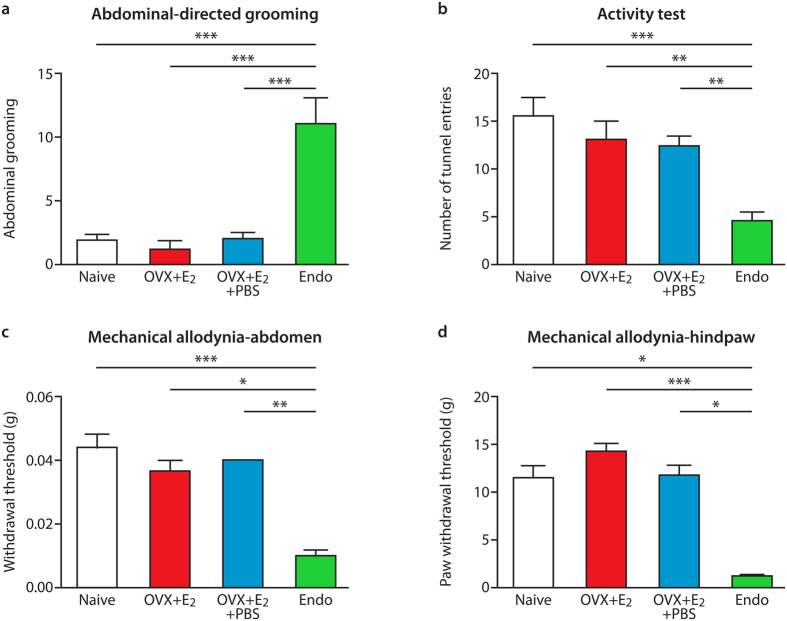
Behavior testing in control and endometriosis mice. Endometriosis mice, together with ovariectomised, estradiol-treated sham recipients, with or without i.p injection of PBS, and naïve controls were scored for general behaviors indicative of discomfort that might be associated with pelvic pain. (**a**) Shows abdominally directed licking (average number of grooming events recorded by two observers over two 5 min periods) was significantly increased in endometriosis mice (n = 5) compared to estradiol-treated (OVX + E_2_; n = 3, OVX + E_2_ + PBS; n = 6) and naïve controls (n = 7). (**b**) Shows that exploratory activity (average number of open-field tunnel entries recorded by two observers was significantly reduced in endometriosis mice (n = 9) compared to estradiol-treated (OVX + E_2_; n = 6, OVX + E_2_ + PBS; n = 6) and naïve controls (n = 11). Mechanical withdrawal threshold, shown by von Frey filament testing, was also measured on the lower abdomen and plantar hind-paw of endometriosis mice (n = 6), OVX + E_2_, OVX + E_2_ + PBS (n = 6) and naïve controls (n = 7). For quantitative sensory testing, von Frey filaments were applied to caudal regions of the abdomen; (**c** and **d**) show that withdrawal thresholds (g = grams) for both abdomen and paw testing were significantly decreased in endometriosis mice compared to the other groups. Statistical analysis was performed using a one-way ANOVA and Newman-Keuls post-hoc test (**a** and **b**) or a Kruskall-Wallis test and Dunn’s multiple comparison test. *p < 0.05. **p < 0.01, ***p < 0.001.

We confirmed lesions in 90% of endometriosis mice (Endo) and those that we did not recover lesions from were excluded from our analysis. The average number of lesions recovered was 1.9 (Supplementary Table 1). Endo mice had significantly higher levels of abdominally-directed licking (Fig. 1a) and decreased exploratory activity (Fig. 1b) compared to controls (p < 0.001). Endo mice had significantly lower mechanical withdrawal thresholds for von Frey filaments, not only on the abdomen (Fig. 1c) but also on the hind-paws (Fig. 1d; p < 0.05 and p < 0.01, respectively). We found no correlation between mechanical allodynia (hypersensitivity) and number of lesions in mice with endometriosis (Supplementary Fig. 1a,b)

### Over-representation of the PGE_2_ signaling pathway and nociceptive ion channels in endometriosis lesions and in the nervous system of mice with endometriosis

**Figure 2:**
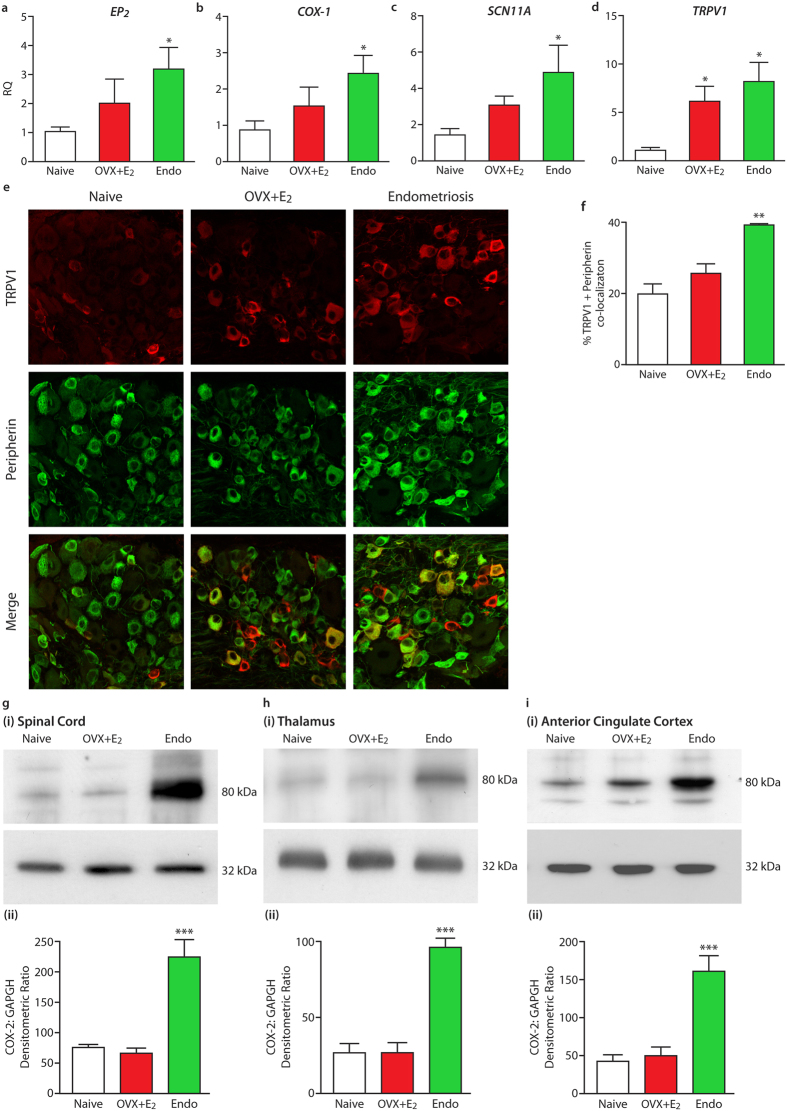
Molecular changes in the nervous system of mice with endometriosis. Expression of the PGE_2_ pathway and nociceptive ion channels is elevated in the DRGs, spinal cord and brain of mice with endometriosis. (**a–d**) QPCR analysis of the prostaglandin E receptor 2(**a**) *EP*_*2*_, (**b**) *COX-1* and nociceptive ion channels (**c**) *SCN11A* and (**d**) *TRPV1* in L5-L6 DRGs from mice with endometriosis (n = 9) compared to naïve (n = 7) and OVX + E_2_ control mice (n = 6). RQ: Relative quantification. Values were normalized to a single naïve DRG sample given the arbitrary value of one. (**e,f**) Dual label immunofluorescence was carried out to identify TRPV1 expression (red) in L5-6 DRG cells co-expressing peripherin (green). (**e**) Shows typical confocal images for TRPV1 and peripherin from naïve, OVX + E_2_-treated sham-recipient and endometriosis mice (field of view, 160 × 160 μm). Total cells counted were 330, 320, and 543, accumulated from three different naïve, OVX + E_2_-treated and endometriosis mice in each case. (**f**) Shows a bar chart that summarizes % expression of TRPV1 in peripherin-positive cells and indicates that the number of TRPV1 + peripherin + small DRG cells is significantly increased in mice with endometriosis. (**g–i**) Images (i) show representative examples of COX-2 (top panel; 80 kDa) and GAPDH (bottom panel; 36 kDa) expression as analyzed using Western blot in (**g**) spinal cord, (**h**) thalamus, (**i**) and anterior cingulate cortex of endometriosis mice, OVX + E_2_-treated sham-recipients and naïve controls, n = 5-6 for all groups. (ii) Graphs showing COX-2: GAPDH densitometric ratio derived from quantitative densitometry of films. Both images and bar charts indicate marked elevation of COX-2 expression in each region. No changes were observed in OVX + E_2_-treated and naïve controls. Statistical analysis was performed using a one-way ANOVA and Tukey’s post comparison test. *p < 0.05, **p < 0.01, ***p < 0.001.

Consistent with our previous studies^22^,^23^,^24^ endometriosis-like lesions were recovered from the walls of the parietal peritoneum and the visceral peritoneum covering the uterus, gut, and intestines; mesentery associated with the gut and intestines; adipose tissue associated with the kidney; and underneath the kidneys. *EP*_*2*_, *EP*_*4*,_ cyclooxygenase-1 (COX-1) and cyclooxygenase-2 (COX-2) were significantly increased in endometriosis lesions compared to the peritoneum of naive mice (NP) or mice with endometriosis (EP; p < 0.01; Supplementary Fig. 2a–d); EP_2_, EP_4_, COX-1 and COX-2 proteins were immunolocalised to glandular and stromal cells in lesions and mesothelial cells in the peritoneum (Supplementary Fig. 2). PGE_2_ concentrations in the peritoneal fluid of Endo mice were significantly increased (p < 0.05; Supplementary Fig. 2E). mRNA concentrations of *EP*_*1*_ and *EP3* were unchanged (Supplementary Fig. 2f,g). *EP*_*2*_, *Cox-1, Scn11a* and *Trpv1* mRNA concentrations were significantly increased in dorsal root ganglia (DRG; clusters of cell bodies of afferent sensory neurons that transmit noxious stimuli from the periphery to the spinal cord) from endometriosis mice (p < 0.05; Fig. 2a–d); *EP*_*4*_ and *Cox-2* were unchanged (Supplementary Fig. 3c,d). *Trpv1* mRNA was also increased in the DRGs from OVX + E_2_ mice. Dual immunofluorescence showed that in small DRG cells expressing peripherin (a marker for small unmyelinated sensory neurons; C-fibre nociceptors), the proportion that were immunopositive for TRPV1 was significantly increased in Endo mice compared to naïve and OVX + E_2_-treated controls (p < 0.05; Fig. 2e,f). The proportion of TRPV1-immunopositive neurons in DRG that express EP_2_ was also increased in these animals (Supplementary Fig. 3e,f). Mice with endometriosis had significantly increased concentrations of COX-2 protein in the spinal dorsal horn (p < 0.001), the thalamus (p < 0.001) and the anterior cingulate cortex of the brain (p < 0.001; Fig. 2f,i). All of these changes would be expected to contribute to pain hypersensitivity.

### Pre-clinical testing and stratification of potential therapies identified EP_2_ as a key target

**Figure 3:**
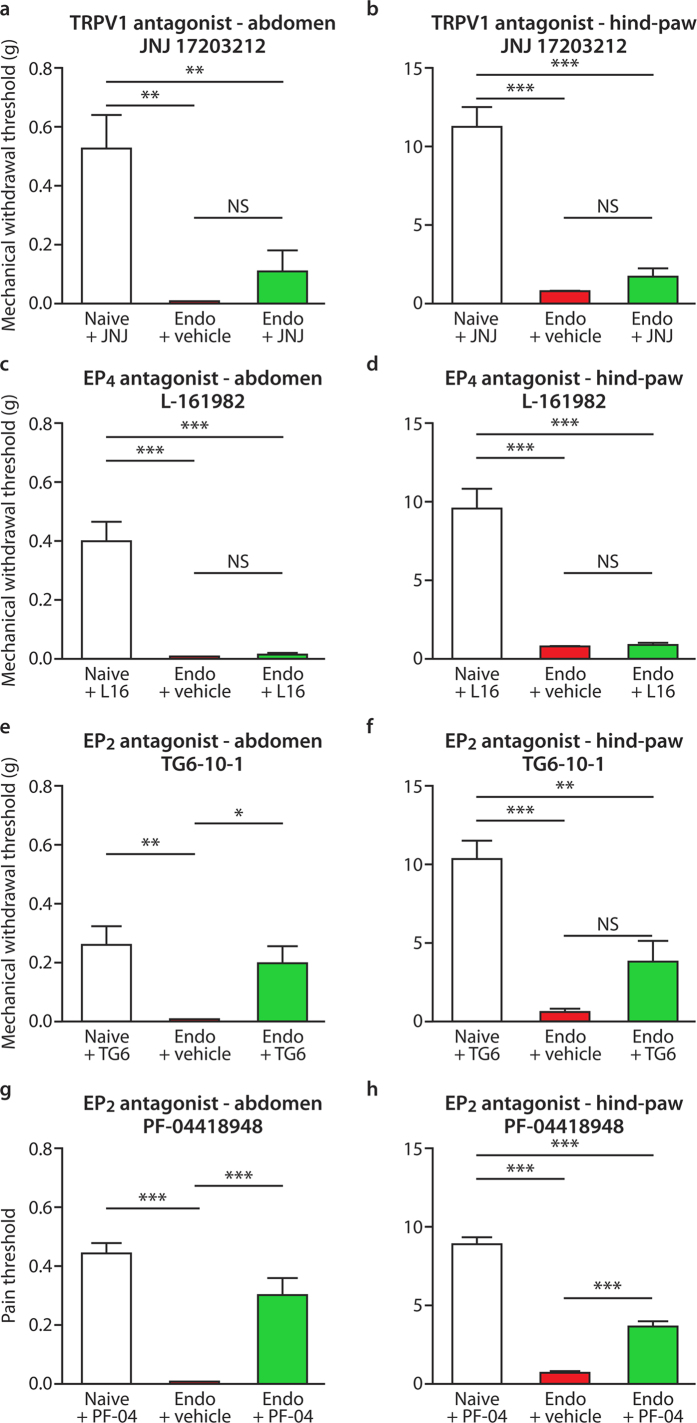
Pre-clinical testing of potential therapeutics in a mouse model of endometriosis. Graphs depict 50% mechanical withdrawal thresholds for von Frey filaments (g = grams) applied to abdomen or hindpaw of mice with endometriosis or naïve controls, n = 5 all groups. The time-point showing maximal reversal of pain is shown for each antagonist. (**a**) Effects of the TRPV1 inhibitor JNJ 17203212 (30 mg/kg ip), 30 mins post-injection on abdominal withdrawal responses in mice with endometriosis compared to naïve mice. (**b**) Shows corresponding results from hindpaw. Concurrently measured naïve values were 0.55 ± 0.17 g and 11.25 ± 1.44 g respectively for abdomen and hindpaw. In both tests, withdrawal thresholds were significantly lower in mice with endometriosis (Endo + Vehicle) than in naïve controls or in naïve animals treated with the drug (Naïve + JNJ; p < 0.01 and p < 0.001). This difference was modestly but not significantly attenuated by JNJ 17203212, which had no discernible effect on the responses of naïve animals. (**c**) Effects of the selective EP_4_ antagonist L-161982 (10 mg/kg ip) 30 mins post i.p injection on abdominal and (**d**) hindpaw mechanical withdrawal thresholds. Naïve values were 0.39 ± 0.07 g and 9.43 ± 1.43 g. In both tests, thresholds were significantly lower in mice with endometriosis than in naïve animals treated with the drug (p < 0.001). L-161982 had no discernible effect on hypersensitivity in mice with endometriosis or in naïve animals. (**e**) Effect of the EP_2_ antagonist TG6-10-1 (10 mg/kg, ip), on abdominal and (**f**) hindpaw mechanical withdrawal thresholds at 45 mins post i.p injection. Naïve values were 0.31 ± 0.11 g and 9.69 ± 1.62 g. In both tests, thresholds were significantly lower in mice with endometriosis compared to naïve controls or in naïve animals treated with the drug (p < 0.01 and p < 0.001). This difference was significantly reversed by TG6-10-1 in abdominal tests (p < 0.05). TG6-10-1 also induced a modest but not statistically significant pain reversal in paw tests. The highly selective EP_2_ antagonist PF-04418948 (10 mg/kg, i.p) significantly attenuated *both* (**g**) abdominal and (**h**) hindpaw mechanical hypersensitivity in mice with endometriosis (p < 0.001) 45 mins post injection. There was no discernible effect of PF-04418948 in naïve animals. Naïve values were 0.48 ± 0.05 g and 8.77 ± 0.51 g. Statistical analysis was performed using a one-way ANOVA and Tukey’s post comparison test. *p < 0.05, **p < 0.001, ***p < 0.001.

**Figure 4:**
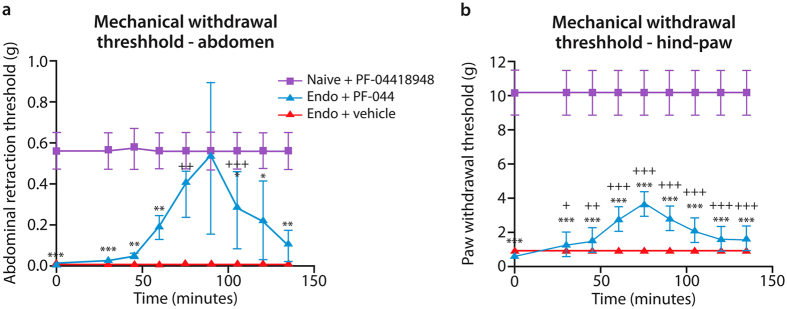
Oral administration of the selective EP_2_ antagonist PF-04418948. To test the efficacy of oral administration of PF-04418948, the drug was administered at a dose of 10 mg/kg by oral gavage and testing was initiated after 30 min. The graphs show the time-course of effects of PF-04418948 on mechanical withdrawal responses in mice with endometriosis (blue and pink lines) or naïve controls (purple lines). In both (**a**) the abdomen and (**b**) the hindpaw, withdrawal thresholds were significantly lower in endometriosis mice than in naïve controls (p < 0.001). The pre-drug withdrawal thresholds in the naïve, drug-treated animals, were not discernibly different from those in naïve, untreated animals (0.55 ± 0.06 g for abdomen and 9.00 ± 0.67 g for hindpaw). In each case the hypersensitivity due to endometriosis was substantially and significantly attenuated by PF-04418948 (p < 0.001), n = 5 all groups. Statistical analysis was performed using a Two-Way ANOVA and Dunnett’s multiple comparison test. *p < 0.05, **p < 0.001 and ***p < 0.001 compared to naïve mice + pharmacological agent. ^+^p < 0.05, ^++^p < 0.01 and ^+++^p < 0.001 compared to pre-administration baseline.

Figure 3 indicates that injection of the TRPV1 inhibitor JNJ 17203212 (Fig. 3a,b) or the EP_4_ antagonist L-161982 (Fig. 3c,d), did not reverse abdominal or paw hyperalgesia to a statistically significant extent. Administration of the EP_2_ antagonist TG6-10-1 resulted in a statistically significant reversal of mechanical allodynia as tested on the abdomen (p < 0.05) at 45 mins post administration but this did not reach significance for the hind paw (Fig. 3e,f). Time-course graphs are shown in Supplementary Fig. 4. Results were extended using injection (Fig. 3g,h) or oral administration (Fig. 4a,b) of a second EP_2_ antagonist (PF-04418948)^25^ and this reduced allodynia in both abdomen and hind-paw tests (p < 0.001 using either route). Oral administration resulted in striking, time dependent, and significant impacts (Fig. 4a,b, p < 0.001).

## Discussion

The re-purposing of drugs and the development of novel treatments for endometriosis-associated pelvic pain has been limited by the paucity of accessible pre-clinical models. In this study we tested pain responses in a mouse model of endometriosis that phenocopies interactions between endometriosis lesions and peritoneal tissue^22^,^24^ identified in humans. Endometriosis (Endo) mice exhibited increased levels of abdominally-directed grooming, a reduction in normal exploratory behavior and reduced mechanical withdrawal thresholds on both the abdomen and plantar hind-paw. The rodent models reported by Berkley, and by Levine and Giudice, both report evidence of hyperalgesia^16^,^20^,^26^,^27^. These models involve the artificial implantation of uterine tissue (full thickness i.e. endometrium plus myometrium) onto the mesenteric arteries of the small intestine and the gastrocnemius muscle, respectively. Therefore the microenvironment of the endometriosis lesion created in these models may not closely mirror that of the peripheral lesions in women.

PGE_2_ is increased in the peritoneal fluid of patients with endometriosis^28^, this up-regulation results from induced expression of cyclooxygenase-2 (COX-2) in endometriotic tissue. PGE_2_ is thought to be a key player in the pathophysiology of endometriosis and studies have shown that inhibition of COX-2 decreases survival, migration and invasion of endometriotic cells^13^. The same authors demonstrated that inhibition of EP_2_ and EP_4_ can inhibit the epithelial and stromal cell invasion via suppression of matrix metalloproteinases^29^. Attenuation of PGE_2_ signaling via lipoxin A_4_ can also modulate disease progression by attenuation of pro-inflammatory and angiogenic mediators^30^. A recent study using xenografted endometriotic cell lines in a nude mouse model of endometriosis demonstrated that dual inhibition of EP_2_ and EP_4_ could attenuate mechanical hyperlagesia of the pelvic floor via suppression of pro-inflammatory mediators in dorsal root ganglia^31^, however the authors did not test secondary hyperalgesia or analyze changes in the central nervous system.

In our mouse model we confirmed that the prostaglandin E signaling pathway was over-expressed in the pelvic cavity of our Endo mice and concentrations of PGE_2_ were increased in their peritoneal fluid. Release of PGE_2_ at sites of peripheral inflammation can contribute to pain hypersensitivity by lowering the threshold and enhancing the excitability of nociceptor sensory fibers^32^. This occurs at least in part via EP receptor-mediated activation of intracellular kinases in the nociceptor terminal that causes phosphorylation of the nociceptive ion channel TRPV1^33^,^34^ and an up-regulation of Nav1.9 voltage-gated sodium channel (SCN11A)^35^, which is then transported to peripheral nerve terminals to contribute to increased excitability^7^. We detected a significant increase in expression of EP_2_, COX-1 and both TRPV1 and SCN11A ion channels in the DRGs of mice with endometriosis. This parallels observations of increased COX-1 and COX-2 in TRPV1-positive DRG cells in other models of inflammatory hyperalgesia^36^. TRPV1-immunoreactivity was increased in small, peripherin-positive, nociceptive neurons in DRG of mice with endometriosis and EP_2_ expression was further increased in these cells. All of these findings are consistent with the development of sensory neuron hyperexcitability in our mice. The possibility of some additional role of prostaglandin signaling in non-neuronal cells, as seen in some other pain models^37^ cannot be excluded. Elevated TRPV1 expression in small DRG cells innervating pelvic regions in mice with endometriosis is consistent with our previous findings that TRPV1 mRNA is elevated in peritoneal lesions from women with endometriosis^38^.

In this study, assessment of COX-2 expression in CNS regions within the pain-processing pathway revealed striking increases in expression at spinal, thalamic and cortical levels. COX-2 expression in the CNS is established as a sensitive and responsive biomarker of centralized inflammatory pain^39^,^40^,^41^ and is an important finding consistent with the inflammatory pain and widespread central sensitization as experienced by women with endometriosis. The phenomenon of central sensitization^42^ has been postulated as a key contributor to the co-morbid pain syndromes experienced by women with the condition^4^. Central sensitization is described as a maladaptation of the CNS resulting from continued or repetitive input from nociceptors, in endometriosis it is likely that this input is provided by afferent nerve fibers innervating endometriosis lesions. One of the first steps in this process of central sensitization is an increase in expression of genes encoding neurotrophins, neuropeptides and ion channels critical in sensing and detecting noxious stimuli^8^. In the model of endometriosis generated by Berkley et al, rats exhibit vaginal hyperalgesia^26^ and it is argued by the authors that this finding suggests central sensitization as an underlying factor because spinal segments associated with the induced endometriosis cysts are distant from spinal segments receiving input from the vagina^43^,^44^. Using this same model a decrease in μ-opioid and NMDA receptor immunoreactivity in the periaqueductal gray area of the brain was detected in rats with endometriosis compared to controls. Torres-Reverón et al suggested that a decrease in NMDA receptor expression could be an attempt to homeostatically regulate pain perception, whilst a decrease in μ-opioid receptor expression suggests decreased modulatory activity of opioid receptors that could contribute to hyperalgesia in the condition^45^. We have also documented molecular alterations in the CNS of mice with endometriosis and we believe this as an important step change in our understanding of endometriosis-associated pain.

Having demonstrated amplified pain behaviors, we tested therapeutic strategies for reversal of mechanical allodynia. The effects of TRPV1 inhibition were modest, whilst antagonism of EP_2_, particularly following oral administration of the highly selective antagonist PF-04418948 had pronounced effects on peripheral and secondary hyperalgesia. This is consistent with reports that EP_2_ null mice do not develop spinal hyperalgesia following induced peripheral inflammation^46^. In a recent study, the effect of a mixture of EP_2_/EP_4_ antagonists had a modest impact on pelvic floor hyperalgesia in mice which may either reflect the limited specificity of the antagonists tested or use of human endometriotic cell lines (not intact tissue fragments) in recipient mice lacking a full complement of immune cells^31^. In our hands, the selective EP_4_ antagonist L-161982, did not reverse the sensitivity to mechanical stimulation.

In summary, we show that induction of endometriosis in mice with an intact immune system is associated with maladaptation of the CNS, consistent with central sensitization^4^. We have demonstrated striking reversal of both peripheral and secondary hyperalgesia via EP_2_ antagonism. In conclusion, we present evidence that a murine model of endometriosis displaying local and central sensitization can be used for pre-clinical testing of therapeutics for endometriosis-associated pain.

## Methods

### Mouse model of endometriosis

Experiments were performed in accordance with the Guidelines of the Committee for Research and Ethical Issues of the International Association for the Study of Pain. Experiments were performed under licensed approval from the UK Home Office (London). C57BL/6 mice (Harlan Laboratories; Derby, UK) were given access to food and water *ad libitum*, ambient temperature and humidity were 21 °C and 50% respectively. Endometriosis was induced in the mice as previously described^22^. In brief, the endometrium of syngeneic donor mice underwent hormonal manipulation and induction of decidualization using an in-house protocol to model endometrial differentiation, breakdown and repair^47^: detailed analysis of tissue samples has shown that progesterone withdrawal (removal of P4 pellet) results in rapid induction of hypoxia, tissue breakdown and induction of angiogenic genes^47^,^48^. Endometrial tissue (~6 hours post-progesterone withdrawal) was recovered by opening the horn and scraping with a scalpel. Approximately 40 mg tissue (equivalent to one decidualized horn) was suspended in 0.2 ml PBS and injected into the peritoneal cavity of ovariectomised recipient mice that were supplemented with 500 ng Estradiol Valerate (EV). This supplementation was maintained by subcutaneous injection of 500 ng EV every 3 days (modification of previously published model). After allowing lesions to form over 21 days behavioral assessments were performed.

### Behavioral assessments

All behavioral tests were performed starting 21 days after endometriosis induction on 2 consecutive days (day 21 and 22). Mechanical allodynia was measured using calibrated Semmes-Weinstein von Frey filaments (Stoelting, Wood Vale, IL), according to the manufacturer’s instructions. Von Frey filaments were applied to the skin perpendicular to the plantar surface of the hindpaws or to the lower abdomen, as in refs 49, 50, 51. Filaments were applied to the abdomen or hind-paw ten times, force in grams (g) of the filament evoking a withdrawal response in 50% of cases was recorded. Initial testing of the abdomen in naïve mice indicated lower thresholds in more caudal regions, in agreement with a previous report^50^. For the following spontaneous behavior tests mice were placed in observation boxes for two 5 min periods and manually observed by two independent investigators (one blinded to experimental group). Periods of spontaneous abdominally directed licking (an element of normal grooming behavior^52^) were recorded and an average generated. Excessive abdominally-directed licking has been reported to represent a useful biomarker of abdominal visceral pain^46^. Each time a mouse exhibited abdominal grooming was recorded as an event. The matrix of brain regions activated during pain includes areas impacting on affective behaviors, corresponding to the anxiety- and depression-like signs associated with chronic pain states^53^. Paradigms of altered affective sate in rodent pain models include reduced exploratory behavior^54^. Exploratory activity here was recorded in a modification of the open-field setting^55^,^56^, with mice retained within their home box with a cardboard tunnel in the centre of the enclosure; open-field tunnel entries were manually recorded by two independent investigators (one blinded to experimental group).

### Experimental groups and sample collection

Four groups of mice were analysed; (i) naïve controls (no surgical procedures; n = 9), (ii) OVX + E_2_ controls (n = 9), for which, mice were ovariectomised and given E_2_ valerate (Sigma, UK), s.c. 500 ng in sesame oil every 2 days to mirror the surgical and hormonal status of the Endo mice; (iii) OVX + E_2_ + PBS controls (n = 6); as in group ii plus i.p injection of PBS, to mirror injection of tissue as in Endo mice (iv) endometriosis mice (Endo mice; as group iii, with ‘menstrual’ donor material in PBS injected i.p (n = 18)). On day 23 mice were culled and the following samples recovered: peritoneal fluid (PF, recovered as in ref. 57 by injecting 3 ml ice cold PBS into the peritoneal cavity followed by gentle massage and recovery (approximately 2 ml was recovered from the injected 3 ml). PF was then centrifuged and frozen), peritoneal biopsy, endometriotic lesions, L5-L6 DRGs, lumbar spinal cord, thalamus and anterior cingulate cortex. Samples were collected into RNAlater (Applied Biosystems, Warrington, UK) and frozen, neutral-buffered formalin prior to paraffin embedding for immunohistochemical analysis (uterus, peritoneum, and endometriosis lesions) or frozen on dry ice prior to sectioning and immunofluorescence staining (DRG) or protein extraction for Western blot analysis (spinal cord and brain). Endometriotic lesions were recognized as red, brown or white tissue deposits on the visceral or parietal peritoneum and were carefully dissected away from any surrounding fat or peritoneum. The presence of glands plus stroma in suspected lesions were confirmed by haematoxylin/eosin staining. Biopsies that did not contain both glands and stroma were not included in further analysis.

### Antagonists

Agents for i.p. injections were dissolved in 10% dimethylsulphoxide, 50% PEG-400, 40% de-ionised H20 and injected in a volume of 100 μl/25 g using 30 mg/kg JNJ 17203212 (TRPV1 antagonist), 10 mg/kg TG6-10-1 (EP_2_ antagonist; Calbiochem)^58^, 10 mg/kg PF-04418948^25^ (EP_2_ antagonist; Abcam),10 mg/kg L-161982 (EP_4_ antagonist; Abcam). Von Frey testing was performed at 30, 45 and 60 minutes post injection: for PF-0418948, testing was also carried out at 75 minutes). PF-04418948 (10 mg/kg in 0.5% w/v methylcellulose +0.1% V/V Tween-20 in purified water) was also administered as an oral gavage and the von Frey test performed every 15 mins starting at 30 mins.

### Quantitative real time PCR

RNA was extracted from control uterine biopsies recovered from naïve mice, peritoneal biopsies from naïve and endometriosis mice, endometriosis lesions and dorsal root ganglia using an RNeasy kit (QIAGEN) according to the manufacturers instructions. RNA was quantified using a NanoDrop ND 1000. Quantitative PCR was performed as detailed in refs 59,60; briefly, cDNA was synthesized using SuperScript VILO enzyme (Invitrogen) with 100ng starting template. PCRs were performed using Roche Universal Probe Library (Roche Applied Science) using primer sequences detailed in Supplementary Table 2. 18S was used as a reference gene. Thermal cycling was performed on a 7900 Fast real-time PCR machine. Data was analysed with RQ manager software (Applied Biosystems) using the ∆∆Ct method; samples were normalised to a uterine control sample.

### Immunodetection

Single antigen immunohistochemistry was performed according to standard protocols^47^,^61^ with citrate antigen retrieval.

#### Dual immunofluorescence

Dorsal root ganglia (DRGs) were embedded in OCT (CellPath) and frozen on dry ice. Sixteen μm cryostat sections were blocked for 1 hour at room temperature then incubated with primary antibodies (Supplementary Table 3). Specificity of the TRPV1 antibody has been previously established^62^. Secondary antibodies, from Molecular Probes or Sigma-Aldrich, goat anti-chicken Alexafluor 488 (1:1000), goat anti-guinea pig AlexaFluor 568 (1:1000) or goat anti-guinea-pig CF405A (1:1000) were applied for 1 hour. Sections were mounted in ProLong® Gold Antifade (Life Technologies). Confocal images were acquired at x20 magnification using a Nikon A1R microscope and ImageJ software was used to quantify co-staining. Standard controls omitting primary antibodies were immunonegative.

### Prostaglandin E_2_ (PGE_2_) ELISA

Approximately 2 ml of the PF was collected into tubes containing indomethacin (10 μM) to prevent *ex vivo* PGE_2_ metabolism. PGE_2_ levels were analyzed using DetectX^®^ prostaglandin E_2_ enzyme immunoassay kit (Arbor Assays, MI, USA).

### Western blotting

Tissue samples were collected into sealable tubes and frozen on dry ice, and then subsequently homogenized in Laemmli buffer, heated to 80 °C for 5 min and centrifuged. Aliquots of lysate supernatant were analysed using the NuPage XCell *Sure*Lock^TM^ Minicell gel electrophoresis system (Invitrogen) with approximately 12 μg protein loaded per lane. Membranes were incubated overnight at 4 °C in 2% non-fat dried milk in 0.1 M PBS with 0.1% Tween-20, containing anti-COX-2 antibody (Supplementary Table 3)^63^. Membranes were washed and incubated for 50 min at room temperature with peroxidase-conjugated donkey anti-rabbit antibody (Chemicon, 1:20,000) and detected by peroxidase-linked enhanced chemiluminescence. Membranes were re-probed with mouse monoclonal anti-GAPDH (Supplementary Table 2). Films were scanned and band intensities were quantified by densitometry using ImageJ.

### Statistical analysis

Statistical analysis used a one-way ANOVA with a Newman Keuls or Tukey’s test, or a Kruskal Wallis with a Dunn’s multiple comparison test. A p value of less than 0.05 was considered significant. *p < 0.05, **p < 0.01, ***p < 0.001.

## Additional Information

**How to cite this article**: Greaves, E. *et al*. EP_2_ receptor antagonism reduces peripheral and central hyperalgesia in a preclinical mouse model of endometriosis. *Sci. Rep.* **7**, 44169; doi: 10.1038/srep44169 (2017).

**Publisher's note:** Springer Nature remains neutral with regard to jurisdictional claims in published maps and institutional affiliations.

## Supplementary Material

Supplementary Information
