# Supplementary material for: EP2 receptor antagonism reduces peripheral and central hyperalgesia in a preclinical mouse model of endometriosis
Source: Sci Rep. 2017 Mar 10;7:44169. doi: 10.1038/srep44169 (PMC5345039; doi:10.1038/srep44169)
Supplement: Supplementary Information [file srep44169-s1.pdf]

EP_2 receptor antagonism reduces peripheral and central hyperalgesia in a preclinical mouse model of endometriosis

Erin Greaves, Andrew W Horne, Helen Jerina, Marta Mikolajczak, Lisa Hilferty, Rory Mitchell, Sue M Fleetwood-Walker, Philippa TK Saunders

Supplementary Information

Supplementary Tables

Supplementary Table 1; Information about Lesions Recovered from the Mouse Model

Sample size	**22**
Recovery rate	90%
Number of lesions (mean±SEM)	1.9±0.22

Supplementary Table 2: Primer sequences.

Gene	Forward	Reverse	UPL Probe
EP_2	5’-cagaggagacggaccacct-3’	5’-ccatgtaggcaaagattgtgaa-3’	2
EP_4	5’-cctaaccccaccctacaggt-3’	5’ agaaggacgcgttgactcc-3’	77
COX-1	5’-cctctttccaggagctcaca-3’	5’-tcgatgtcaccgtacagctc-3’	46
COX-2	5’-gatgctcttccgagctgtg-3’	5’-ggattggaacagcaaggattt-3’	45
TRPV1	5’-caacaagaaggggcttacacc-3’	5’-tctggagaatgtaggccaagac-3’	21
SCN11A	5’-ttcataatgtgtggcaactgg-3’	5’-ttattgcacgtggaaccatc-3’	29

Supplementary Table 3: Antibodies.

Antibody	Raised in	Application	Supplier	Dilution
EP_2	Rabbit	Immunohistochemistry	Cayman; 101750	1:100
EP_4		Immunohistochemistry	Cayman;101775	1:50
COX-1		Immunohistochemistry	Bio-Vision; 3361-100	1:25
COX-2 (H-62)	Rabbit	Immunohistochemistry	Santa Cruz Biotech; sc-7951	1:25
TRPVI	Guinea pig	Dual immunofluorescence	Abcam; ab10295	1:1000
Peripherin	Chicken	Dual immunofluorescence	Abcam; ab39374	1:5000
NF-200	Chicken	Dual immunofluorescence	Millipore; SS39	1: 15000
EP_2	Rabbit	Dual Immunofluorescence	Alomone; APR-064	1:1000

COX-2	Rabbit	Western blot	Santa-Cruz Biotech; sc-7951	1:200
GAPDH	Mouse	Western blot	Millipore; MAB 374	1:10000

Supplementary Table 3. It has been reported that the expression in mouse uterus of standard “housekeeping genes” such as GAPDH can be altered by E_2 treatment (Schroder et al., 2009). However, in the CNS regions assessed here, the OVX + E_2 and endometriosis model groups showed no discernible changes in GAPDH expression compared to naïve mice. For equal gel loading volumes of lysates produced at a fixed ratio of tissue wet weight per ml, we found densitometric measurements of GAPDH expression in the OVX + E_2 group to be 112.9 ± 11.9%, 101.3 ± 6.2% and 107.3 ± 6.4% of naïve for spinal cord, thalamus and anterior cingulate cortex respectively (means ± SEM, n = 5-6). Corresponding values for the endometriosis model group were 96.6 ± 6.2%, 96.5 ± 5.4% and 95.4 ± 6.5%. Analysis by One-Way ANOVA with Dunnett’s post-hoc test indicated no statistically significant differences in any case, validating our use of GAPDH here as a comparator “housekeeping gene”.

Supplementary Figures

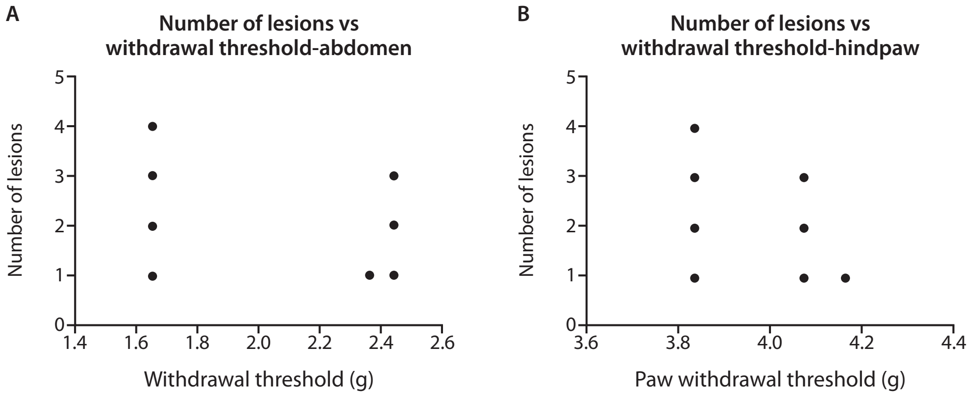

Fig.S1| Endometriosis mice show no correlation between mechanical hyperalgesia and number of lesions. Correlation between withdrawal threshold (g) and number of lesions when von Frey filaments were applied to a) the abdomen and b) the hindpaw. N=10 mice.

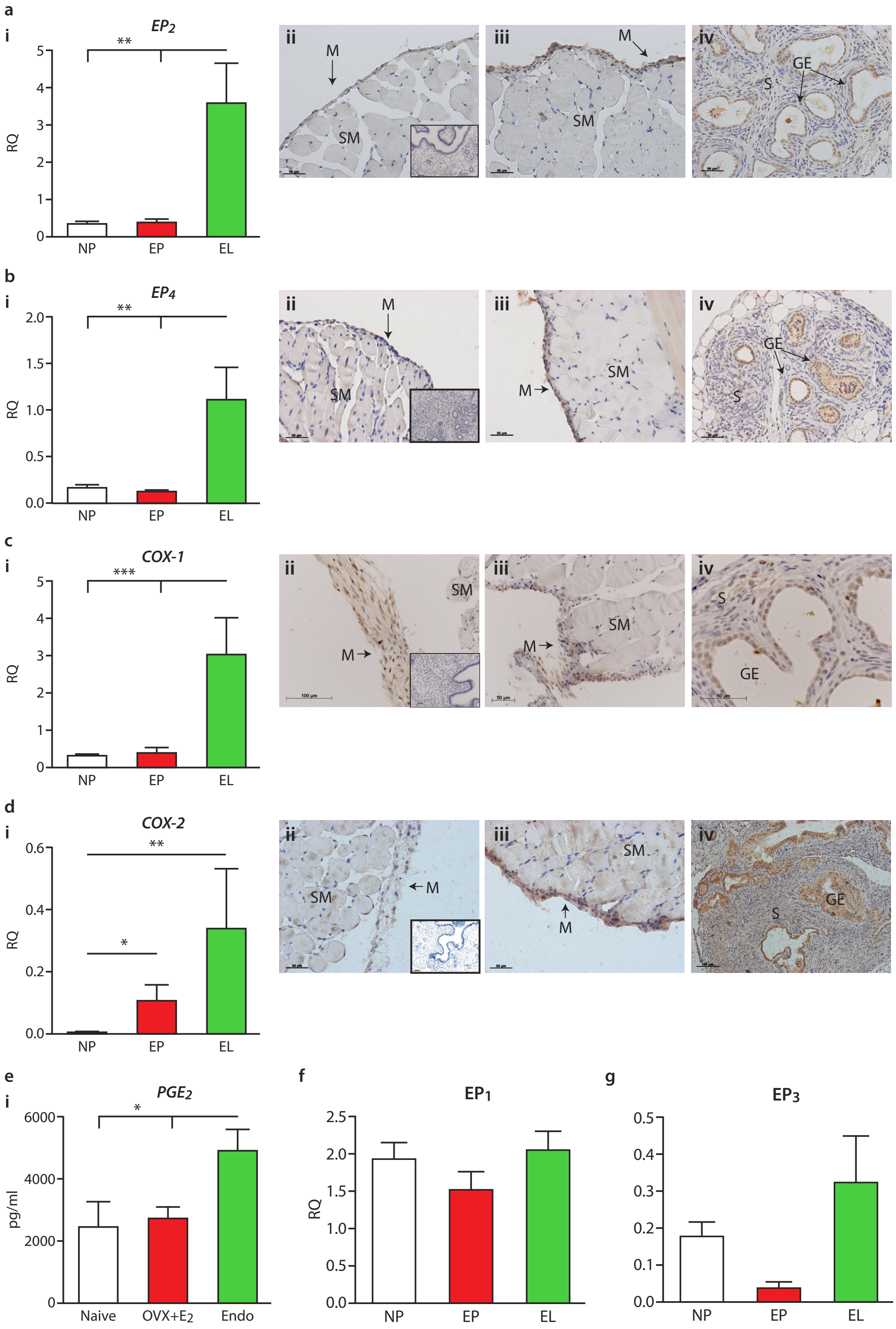
a
i
EP2
**
RQ
NP
EP
EL
ii
iii
iv
M
SM
GE
S
b
i
EP4
**
RQ
c
i
COX-1
***
d
i
COX-2
**
*
e
i
PGE2
*
pg/ml
Naive
OVX+E2
Endo
f
EP1
g
EP3

Fig.S2│The PGE_2 signaling pathway is over-expressed in endometriosis lesions. mRNA concentrations and protein expression of **(a)** EP_2, **(b)** EP_4, **(c)** COX-1, **(d)** COX-2 in peritoneum from naïve mice (NP; ii), peritoneum from mice with endometriosis (EP; iii) and endometriosis lesions (EL; iv). (i) Graphs indicate QPCR data where values are normalized to expression in samples of cycling uterus. NP, n=9, EP, n=6 and EL, n=8. RQ: Relative quantification. (ii-iii) Representative images of single antigen immunodetection taken from NP, EP and EL. Insets are negative controls (omission of primary antibody on sections of cycling uterus). Scale bars = 50 or 100 μM. **(e)** Peritoneal fluid concentrations of PGE_2. PGE_2 concentrations (pg/ml) were measured using ELISA in the peritoneal fluid of mice with endometriosis (n=14) compared to naïve (n=8), and OVX+E_2 mice (n=7). **(f-g)** mRNA concentrations of **(f)** EP_1 and **(g)** EP_3 were also analyzed in lesions and peritoneum, there was no significant difference in expression levels and therefore these particular receptors were not investigated further. Statistical analysis was performed using a one-way ANOVA and Newman-Keuls post comparison test. *$p<0.05$, **$p<0.01$, ***$p<0.001$.

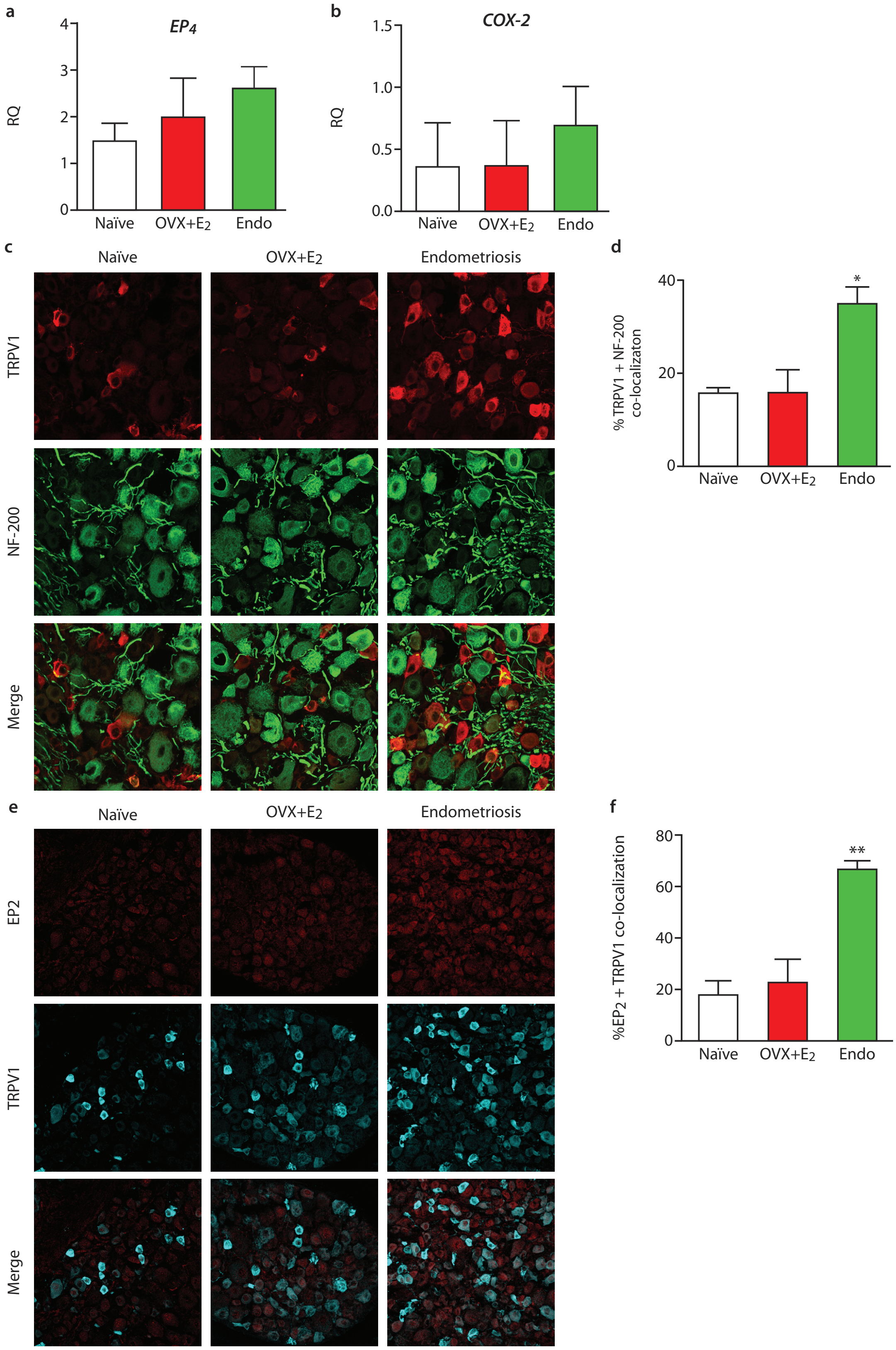

Fig.S3| Gene expression in DRGs of endometriosis mice. (a-b) QPCR analysis of the prostaglandin E receptor **(a)** *EP*$_4$, and **(b)** *COX-2* in L5-L6 DRGs from mice with endometriosis (n=9) compared to

naïve (n=7) and OVX+E_2 control mice (n=6). RQ: Relative quantification. Values were normalized to a single naïve DRG sample given the arbitrary value of one. Means were not significantly different. **(c-d)** Dual label immunofluorescence was carried out to identify TRPV1 expression (red) in L5-6 DRG cells co-expressing neurofilament-200 (NF-200; green). **(c)** Shows typical confocal images for TRPV1 and NF-200 from naïve, OVX+E_2 and endometriosis mice (field of view, 160x160µm). Total cells counted were 142, 168, and 204, accumulated from three different naïve, OVX+E_2-treated and endometriosis mice in each case. **(d)** Shows a bar chart that summarizes % expression of TRPV1 in NF-200-positive cells and indicates that the number of TRPV1+NF-200+ small DRG cells is significantly increased in mice with endometriosis. Statistical analysis was performed using a one-way ANOVA and Tukey's post-hoc test. *$p<0.05$. **(e-f)** Dual label immunofluorescence was carried out to identify EP_2 expression (red) in L5-6 DRG cells co-expressing TRPV1 (cyan). **(e)** Showes typical confocal images for EP_2 and TRPV1 from naïve, OVX+E_2 and endometriosis mice (field of view, 160x160µM). Total cells counted were 119, 58, 206, accumulated from three different naïve, OVX+E_2-treated and endometriosis mice in each case. **(f)** Shows a bar chart that summarises % expression of EP_2 in TRPV1-positive cells and indicates that the number of EP_2+, TRPV1+ small DRG cells is significantly increased in endometriosis. Statistical analysis was performed using a one-way ANOVA and Tukey's post-hoc test. **$p<0.01$.

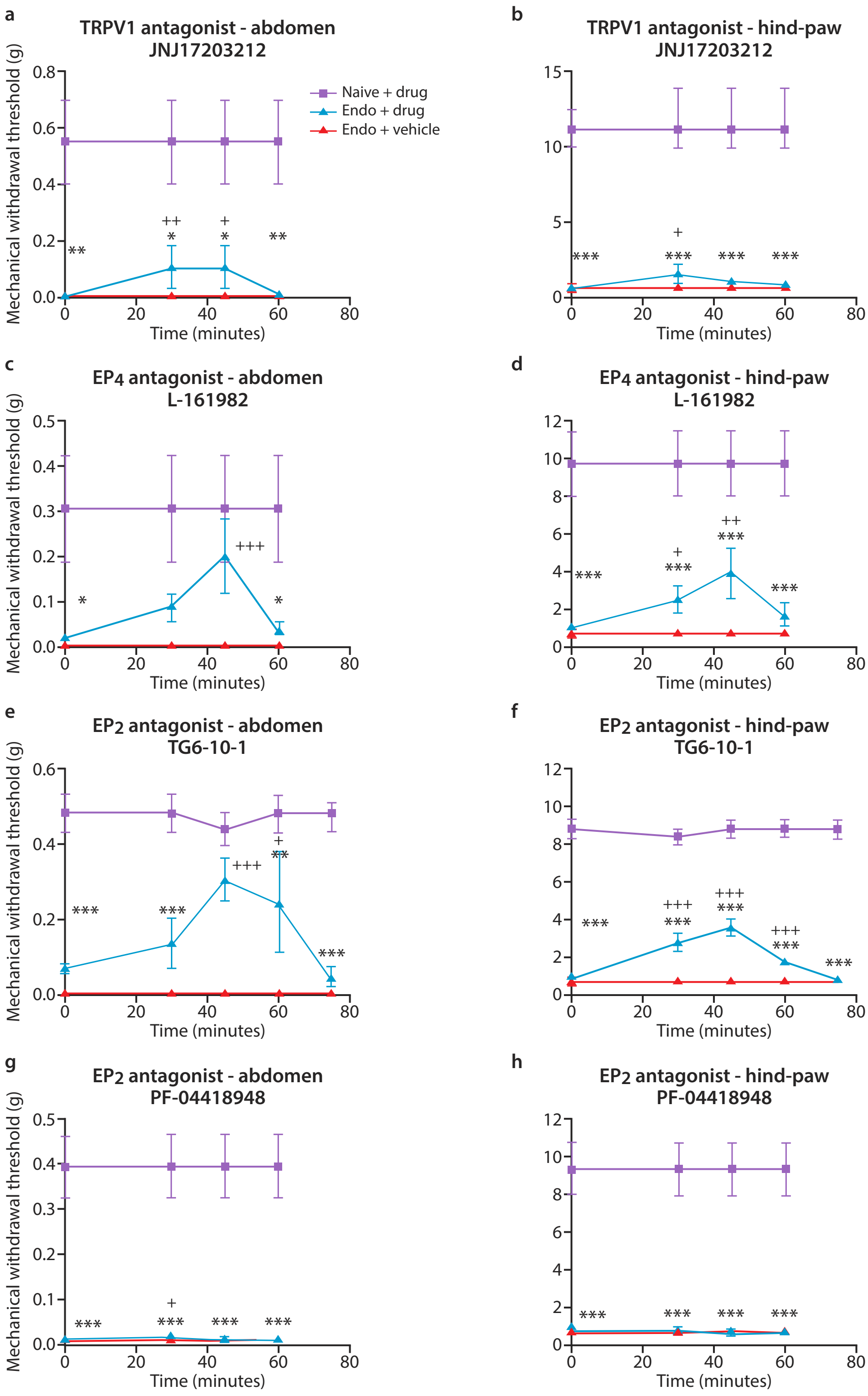

Fig.S4 │ Time-course of effects on local and secondary mechanical hyperalgesia of i.p injection of potential therapeutics in a mouse model of endometriosis. Graphs depict withdrawal thresholds for von Frey filaments applied to the rostral regions of the abdomen or hindpaws of endometriosis mice

compared to naïve controls. OVX+E_2 mice were not tested because Figure 1 indicated that their responses were unaltered from naïve control mice. **(a)** Shows the time-course of effects of the TRPV1 inhibitor JNJ 17203212 (30 mg/kg ip) on abdominal and **(b)** hindpaw mechanical withdrawal responses in endometriosis mice (blue and pink lines) or naïve controls (purple lines). In both abdomen and paw tests, withdrawal thresholds were significantly lower in endometriosis mice than in naïve mice ($p<0.01$ and $p<0.001$). These differences were modestly but significantly attenuated with JNJ 17203212 ($p<0.01$and $p<0.05$), which had no discernible effect in naïve controls. **(c)** Shows the time-course effects of the EP_2 antagonist TG6-10-1 (10mg/kg, ip), on abdominal and **(d)** paw mechanical withdrawal threshold in endometriosis mice compared to naïve controls. In both abdomen and paw tests, withdrawal thresholds were significantly lower in endometriosis mice compared to naïve controls ($p<0.05$ and $p<0.001$). These differences were substantially and significantly attenuated by TG6-10-1 ($p<0.001$ and $p<0.01$). TG6-10-1 had no discernible effect in naïve controls. The highly selective EP_2 antagonist PF-04418948 (10mg/kg, ip) substantially and significantly reversed **(e)** abdominal and **(f)** hindpaw mechanical hyperalgesia ($p<0.001$). There was no discernible effect of PF-04418948 in naïve mice. The selective EP_4 antagonist L-161982 had only a very small but statistically significant effect on **(g)** abdominal mechanical hyperalgesia and no discernible effect on **(h)** paw mechanical hyperalgesia (n=5 in all cases). Statistical analysis was performed using a Two-Way ANOVA with a Dunnett's multiple comparison test. *$p<0.05$, **$p<0.001$ and ***$p<0.001$ compared to naïve mice + pharmacological agent. +$p<0.05$, ++$p<0.01$ and +++$p<0.001$ compared to pre-administration baseline.
